# Association of Respiratory Viruses with Outcomes of Severe Childhood Pneumonia in Botswana

**DOI:** 10.1371/journal.pone.0126593

**Published:** 2015-05-14

**Authors:** Matthew S. Kelly, Marek Smieja, Kathy Luinstra, Kathleen E. Wirth, David M. Goldfarb, Andrew P. Steenhoff, Tonya Arscott-Mills, Coleen K. Cunningham, Sefelani Boiditswe, Warona Sethomo, Samir S. Shah, Rodney Finalle, Kristen A. Feemster

**Affiliations:** 1 Botswana-UPenn Partnership, Gaborone, Botswana; 2 Global Health Center, The Children’s Hospital of Philadelphia, Philadelphia, Pennsylvania, United States of America; 3 Division of Infectious Diseases, The Children’s Hospital of Philadelphia, Philadelphia, Pennsylvania, United States of America; 4 Division of Pediatric Infectious Diseases, Duke University Medical Center, Durham, North Carolina, United States of America; 5 Department of Pathology and Molecular Medicine, McMaster University, Hamilton, Ontario, Canada; 6 Department of Epidemiology, Harvard T.H. Chan School of Public Health, Boston, Massachusetts, United States of America; 7 Perelman School of Medicine, University of Pennsylvania, Philadelphia, Pennsylvania, United States of America; 8 University of Botswana School of Medicine, Gaborone, Botswana; 9 Divisions of Hospital Medicine and Infectious Diseases, Cincinnati Children’s Hospital Medical Center, Cincinnati, Ohio, United States of America; Kliniken der Stadt Köln gGmbH, GERMANY

## Abstract

**Background:**

The highest incidence of childhood acute lower respiratory tract infection (ALRI) is in low- and middle-income countries. Few studies examined whether detection of respiratory viruses predicts ALRI outcomes in these settings.

**Methods:**

We conducted prospective cohort and case-control studies of children 1-23 months of age in Botswana. Cases met clinical criteria for pneumonia and were recruited within six hours of presentation to a referral hospital. Controls were children without pneumonia matched to cases by primary care clinic and date of enrollment. Nasopharyngeal specimens were tested for respiratory viruses using polymerase chain reaction. We compared detection rates of specific viruses in matched case-control pairs. We examined the effect of respiratory syncytial virus (RSV) and other respiratory viruses on pneumonia outcomes.

**Results:**

Between April 2012 and August 2014, we enrolled 310 cases, of which 133 had matched controls. Median ages of cases and controls were 6.1 and 6.4 months, respectively. One or more viruses were detected from 75% of cases and 34% of controls. RSV and human metapneumovirus were more frequent among cases than controls, but only enterovirus/rhinovirus was detected from asymptomatic controls. Compared with non-RSV viruses, RSV was associated with an increased risk of treatment failure at 48 hours [risk ratio (RR): 1.85; 95% confidence interval (CI): 1.20, 2.84], more days of respiratory support [mean difference (MD): 1.26 days; 95% CI: 0.30, 2.22 days], and longer duration of hospitalization [MD: 1.35 days; 95% CI: 0.20, 2.50 days], but lower in-hospital mortality [RR: 0.09; 95% CI: 0.01, 0.80] in children with pneumonia.

**Conclusions:**

Respiratory viruses were detected from most children hospitalized with ALRI in Botswana, but only RSV and human metapneumovirus were more frequent than among children without ALRI. Detection of RSV from children with ALRI predicted a protracted illness course but lower mortality compared with non-RSV viruses.

## Introduction

Globally, an estimated 150 million episodes of acute lower respiratory infection (ALRI) occur each year among children [1]. Most of these infections are caused by respiratory viruses, particularly in children under two years of age. Polymerase chain reaction (PCR)-based assays identify one or more viruses in 50–85% of pediatric ALRI episodes [2–9]. However, determining the clinical significance of detection of respiratory viruses from children using PCR can be challenging. Virus-virus coinfections and mixed viral-bacterial infections occur in 15–30% of cases [5,10], and viruses can be detected in 25–45% of children in the absence of respiratory symptoms [5–7].

The incidence of pediatric ALRI is highest in low- and middle-income countries (LMICs) [1]. In sub-Saharan Africa alone, more than 35 million episodes occur each year, resulting in 500 000 deaths and straining of the health care systems of many of the world’s poorest countries [1]. Real-time multiplex PCR is appealing for low-resource settings because this technology can be highly automated and is thus less laborious and expensive than conventional methods [11]. Recent studies using real-time PCR confirmed the substantial role played by respiratory viruses in pediatric ALRI episodes in LMICs [6–9]. However, few studies examined whether detection of respiratory viruses by PCR can predict ALRI outcomes in these settings.

We conducted prospective cohort and case-control studies to identify the viral etiologies of severe ALRI among infants and young children in Botswana, and examined whether identification of respiratory syncytial virus (RSV) and non-RSV viruses by PCR is associated with ALRI outcomes.

## Methods

### Setting

These studies were conducted in Gaborone, Botswana between April 2012 and August 2014. Botswana has a semi-arid climate, with a short rainy season that typically occurs from November to March. The country’s HIV prevalence among adults aged 15–49 years is 21.9% [12]. *Haemophilus influenzae* type B (Hib) conjugate vaccine was included in the country’s immunization schedule in 2010. Pneumococcal conjugate vaccine (PCV-13) was introduced in July 2012. The public sector operates 98% of health facilities in Botswana, including an extensive network of clinics and health posts in the Gaborone area [13].

### Prospective Cohort Study

We conducted a hospital-based, prospective cohort study to identify the viral etiologies of ALRI in Botswana and investigate their association with ALRI outcomes. Eligible children were 1–23 months of age presenting to a tertiary hospital with pneumonia, defined by the World Health Organization (WHO) as cough or difficulty in breathing with lower chest wall indrawing [14]. Children with one or more danger signs (central cyanosis, convulsions, inability to drink, or abnormal sleepiness) were classified as having severe pneumonia [14]. We excluded children with a chronic medical condition predisposing to pneumonia (other than HIV), hospitalization in the prior 14 days, asthma, or wheezing with resolution of lower chest wall indrawing after ≤2 bronchodilator treatments. All children were recruited within six hours of the triage time in the Emergency Department, and clinical care was provided by medical officers and pediatric residents on a ward supervised by pediatric specialists. Departmental guidelines for treatment of pneumonia were based on WHO recommendations, but antibiotic treatment decisions were ultimately at the discretion of the supervising pediatrician [14].

Sociodemographic and clinical data were collected at enrollment from physical examination, infant and maternal medical records, and a face-to-face interview with the child’s caregiver(s). Research staff assessed children and reviewed charts daily until hospital discharge (or death). The primary outcome, treatment failure, was assessed at 48 hours by a study physician or nurse blinded to enrollment data. Treatment failure was defined as persistent lower chest wall indrawing, the development of new WHO danger signs, oxygen saturation <80% (on room air), requirement for continuous positive airway pressure (CPAP) or mechanical ventilation, or death. This definition was adapted for our setting from criteria used in a previous study of childhood pneumonia [15], and training sessions were held every three months during the study period to standardize the assessment process. Cases discharged before 48 hours were considered treatment responders but caregivers were contacted by telephone to confirm treatment response. Secondary outcomes included days of respiratory support (supplemental oxygen, CPAP, or mechanical ventilation), length of stay, and in-hospital mortality.

### Case-Control Study

In order to examine the strength of associations between detection of specific respiratory viruses and ALRI, we enrolled in parallel a group of community-based control subjects without ALRI. These controls were children 1–23 months of age receiving well child or acute care services at one of 18 public clinics in the Gaborone area. Controls were matched to pneumonia cases 1:1 by primary care clinic and date (≤2 weeks from the case enrollment). Exclusion criteria were WHO-defined pneumonia, a chronic medical condition predisposing to pneumonia (other than HIV), hospitalization in the prior 14 days, and asthma. Control children with and without symptoms of upper respiratory infection (URI; presence of rhinorrhea, nasal congestion, or cough) were enrolled. As with children enrolled in the pneumonia cohort, we collected enrollment data for controls from physical examination, infant and maternal medical records, and a face-to-face interview with the child’s caregiver(s).

Children could be enrolled more than once as either a case or control subject provided that the second enrollment occurred ≥30 days after hospital discharge for a prior case enrollment or ≥30 days after the enrollment date for a prior control enrollment.

### Laboratory methods

Nasopharyngeal swab specimens were obtained from cases and controls at enrollment using flocked swabs and universal transport media (Copan Italia, Brescia, Italy). These specimens were stored at -80°C and shipped on dry ice at 6-month intervals to the Regional Virology Laboratory (St. Joseph’s Healthcare, Hamilton, ON, Canada). Testing for RSV, influenza viruses A and B, parainfluenza virus types 1–3, human metapneumovirus (hMPV), and adenovirus was performed using real-time multiplex PCR. Testing for enterovirus/rhinovirus (E/R) was conducted via a uniplex PCR assay, and positive samples were further characterized by sequencing of the amplicon to identify the species.

### Statistical analysis

We described baseline characteristics of case and control subjects using frequencies and percentages for categorical variables and medians and interquartile ranges (IQR) for continuous variables. We used chi-square tests conditioning on the matched pairs to compare baseline characteristics of case and control subjects. We used generalized estimating equations to account for correlated data among children enrolled more than once as either a case or control subject. To examine pneumonia outcomes by detection of respiratory viruses, we classified case subjects as having RSV only, RSV and non-RSV virus coinfection, non-RSV viruses only, or no respiratory viruses. We used Cox proportional hazards to directly estimate risk ratios for treatment failure at 48 hours and in-hospital mortality and linear regression to estimate mean differences in days of respiratory support and length of stay in comparing these groups [16]. Adjusted analyses included the following variables identified a priori based on a literature review: age, low birth weight, HIV exposure status, severe malnutrition, current breastfeeding, household use of wood as a cooking fuel, WHO disease severity, and hypoxia (oxygen saturation <90% on room air) at enrollment [17–19]. For the matched case-control study, we used Fisher’s exact test conditioning on the matched pairs to compare the frequency of detection of specific respiratory viruses among cases and controls.

Study data were managed using Research Electronic Data Capture (REDCap) tools hosted at The Children’s Hospital of Philadelphia (Philadelphia, PA) [20]. All statistical analyses were conducted using SAS software version 9.3 (SAS Institute, Cary, NC). These studies were approved by the Health Research and Development Committee (Ministry of Health, Botswana) and institutional review boards at Princess Marina Hospital, the University of Pennsylvania, Duke University, and McMaster University. For both studies, written informed consent was obtained from a legal guardian after a detailed explanation of the study procedures.

## Results

### Prospective Cohort Study

We enrolled 310 children with pneumonia during the study period (Table 1, columns 1 and 2). Median [IQR] age was 6.1 [2.8, 13.3] months, and 55% were male. One hundred and four (34%) children presented with severe pneumonia. One or more respiratory viruses were detected from 232 of 310 (75%) children (Table 2, columns 1 and 2), including RSV only in 89 (29%), RSV and non-RSV virus coinfection in 18 (6%), and non-RSV viruses in 125 (40%). One hundred and six (34%) children failed treatment at 48 hours and 18 (5.8%) died. Median [IQR] length of stay for the 292 children surviving to hospital discharge was 3.8 [1.9, 7.9] days. One-hundred and eighty-five (60%) children received supplemental oxygen, 32 (10%) required CPAP, and five (2%) children were mechanically ventilated during the hospitalization.

**Table 1:** Baseline characteristics of N = 310 children 1 to 23 months of age with pneumonia and N = 133 matched control children in Gaborone, Botswana, April 2012 to August 2014.

	Pneumonia Cases				Matched Controls^a^						
	All (n = 310)^b^		Matched (n = 133)		All (n = 133)		URI^c^ (n = 66)		No URI^c^ (n = 67)		Comparison of Matched Cases and Controls^d^
Characteristic (case, control n with data)	n	%	n	%	n	%	n	%	n	%	*P*
**Demographics**											
Age, months (n = 310, n = 133)											0.40
1–5	154	50	65	49	58	44	20	30	38	57	
6–23	156	50	68	51	75	56	46	70	29	43	
Male gender (n = 310, n = 133)	171	55	76	57	68	51	39	59	29	43	0.32
Birth weight <2500 grams (n = 306, n = 133)	62	20	28	21	24	18	11	17	13	19	0.53
HIV exposure status (n = 306, n = 119)											0.001
HIV-unexposed	193	63	74	57	94	79	46	79	48	79	
HIV-exposed, uninfected	89	29	47	36	25	21	12	21	13	21	
HIV-infected	24	8	9	7	0	0	0	0	0	0	
**Nutrition and infant feeding practices**											
Current breastfeeding (n = 310, n = 128)	126	41	50	38	67	50	31	47	36	54	0.05
Severe malnutrition^e^ (n = 294, n = 0)	25	9	14	11	-	-	-	-	-	-	NA
**Socioeconomic factors**											
Maternal education level (n = 310, n = 133)											0.01
None or primary	35	11	16	12	5	4	5	8	0	0	
Secondary	211	68	92	69	87	65	42	64	45	67	
Tertiary	64	21	25	19	41	31	19	29	22	33	
Electricity in household (n = 310, n = 133)	197	64	70	53	90	68	42	64	48	72	0.01
Municipal or private water source (n = 310, n = 133)	266	86	110	83	123	92	60	91	63	94	0.01
Refrigerator in household (n = 310, n = 133)	184	59	65	49	88	66	42	64	46	69	0.004
Use of wood as a cooking fuel (n = 310, n = 133)	108	35	37	28	22	17	11	17	11	16	0.03
**Vaccination status**											
*Haemophilus influenza* type B (Hib) vaccine (n = 308, n = 133)											0.36
0 doses	55	18	23	17	16	12	3	5	13	19	
1–2 doses	84	27	36	27	34	26	16	24	18	27	
≥3 doses	169	55	74	56	83	62	47	71	36	54	
Pneumococcal conjugate vaccine (n = 308, n = 133)											0.59
0 doses	172	56	59	44	51	38	22	33	29	43	
1–2 doses	64	21	36	27	39	29	21	32	18	27	
≥3 doses	72	23	38	29	43	32	23	35	20	30	

URI, upper respiratory infection; NA, not analyzed; WHO, World Health Organization

^a^Controls were matched to cases 1:1 by primary care clinic and date (≤2 weeks from the case enrollment).

^b^5 children were enrolled as case subjects twice and one child was enrolled as a case subject on three occasions.

^c^Defined as presence of rhinorrhea, nasal congestion, or cough.

^d^Wald χ^2,^ P-values; generalized estimating equations were used to account for correlated data among children enrolled more than once as case subjects.

^e^Weight-for-length <-3 standard deviation on standard WHO growth curves, mid-upper arm circumference <115mm (for children ≥6 months of age), or bilateral edema of nutritional origin.

**Table 2:** Respiratory viruses detected using multiplex PCR of nasopharyngeal specimens from n = 310 pneumonia episodes among children 1 to 23 months of age and n = 133 matched control children in Gaborone, Botswana, April 2012 to August 2014.

	Pneumonia Cases								Matched Controls												
	All (n = 133)				Matched (n = 133)				All (n = 133)				URI^a^ (n = 66)				No URI (n = 67)				Comparison of Matched Cases and Controls^b^
	n		%		n		%		n		%		n		%		n		%		*P*
RSV	107		35		35		26		2		2		2		3		0		0		<0.0001
Influenza virus	11		4		6		5		2		2		2		3		0		0		0.10
A		7		2		3		2		2		2		2		3		0		0	
B		4		1		3		2		0		0		0		0		0		0	
Parainfluenza virus	17		5		7		5		2		2		2		3		0		0		0.10
Type 1		2		1		0		0		0		0		0		0		0		0	
Type 2		3		1		3		2		1		1		1		2		0		0	
Type 3		12		4		4		3		1		1		1		2		0		0	
Human metapneumovirus	20		6		11		8		0		0		0		0		0		0		
Adenovirus	4		1		0		0		0		0		0		0		0		0		
Rhinovirus/enterovirus	97		31		40		30		40		30		26		39		14		21		>0.99
Rhinovirus A		35		11		15		11		16		12		12		18		4		6	
Rhinovirus B		7		2		5		4		3		2		1		2		2		3	
Rhinovirus C		40		13		14		11		12		9		8		12		4		6	
Other		15		5		6		5		9		7		5		8		4		6	
Any virus	232		75		93		70		45		34		31		47		14		21		<0.0001
>1 virus	24		8		6		5		1		1		1		2		0		0		0.03

PCR, polymerase chain reaction; URI, upper respiratory infection; RSV, respiratory syncytial virus; NA, not analyzed

^a^Defined as presence of rhinorrhea, nasal congestion, or cough.

^b^ *P* value estimated using Fisher’s exact test.

Outcomes by detection of RSV and other respiratory viruses are shown in Table 3. Compared with detection of non-RSV viruses, detection of RSV only was associated with treatment failure at 48 hours [risk ratio (RR): 1.85; 95% CI: 1.20, 2.84], a requirement for more days of respiratory support [mean difference (MD): 1.26 days; 95% CI: 0.30, 2.22 days], and longer duration of hospitalization [MD: 1.35 days; 95% CI: 0.20, 2.50 days]. However, in-hospital mortality was significantly lower among children with RSV only than among children with non-RSV viruses [RR: 0.09, 95% CI: 0.01, 0.80]. The outcomes of children with RSV only did not significantly differ from those with negative viral testing, although in-hospital mortality tended to be lower (*P* = 0.13).

**Table 3:** Outcomes according to detection of RSV and other respiratory viruses among children 1 to 23 months of age with pneumonia in Gaborone, Botswana, April 2012 to August 2014.

	n (%) or Median [IQR]		RR or MD (95% CI)^a^		*P*
**RSV vs. non-RSV viruses**					
Treatment failure at 48 hours					
RSV only (n = 89)	40	(45)	1.85	(1.20, 2.84)	0.01
RSV and non-RSV virus coinfection (n = 18)	7	(39)	2.26	(1.06, 4.84)	0.04
Non-RSV viruses (n = 125)	28	(22)	1	Ref	-
Days of O2, CPAP, or mechanical ventilation					
RSV only	2	[0, 4]	1.26	(0.30, 2.22)	0.01
RSV and non-RSV virus coinfection	1	[0, 3]	0.91	(-0.76, 2.58)	0.28
Non-RSV viruses	1	[0, 2]	0	Ref	-
Length of stay, days^b^					
RSV only	5.1	[2.1, 8.0]	1.35	(0.20, 2.50)	0.02
RSV and non-RSV virus coinfection	4.0	[2.1, 9.0]	1.40	(-0.59, 3.39)	0.17
Non-RSV viruses	2.3	[1.2, 5.2]	0	Ref	-
In-hospital mortality^c^					
RSV only	3	(3.4)	0.09	(0.01, 0.80)	0.03
Non-RSV viruses	8	(6.4)	1	Ref	-
**RSV vs. no respiratory viruses**			8	6.40	
Treatment failure at 48 hours					
RSV only (n = 89)	40	(45)	1.29	(0.88, 1.89)	0.19
RSV and non-RSV virus coinfection (n = 18)	7	(39)	1.37	(0.65, 2.88)	0.41
No respiratory viruses (n = 78)	31	(40)	1	Ref	-
Days of O2, CPAP, or mechanical ventilation					
RSV only	2	[0, 4]	-0.06	(-1.94, 1.83)	0.95
RSV and non-RSV virus coinfection	1	[0, 3]	-0.56	(-3.64, 2.52)	0.72
No respiratory viruses	2	[0, 6]	0	Ref	-
Length of stay, days^b^					
RSV only	5.1	[2.1, 8.0]	-0.49	(-2.36, 1.39)	0.61
RSV and non-RSV virus coinfection	4.0	[2.1, 9.0]	-0.39	(-3.41, 2.62)	0.80
No respiratory viruses	5.1	[2.5, 13.0]	0	Ref	-
In-hospital mortality^c^					
RSV only	3	(3.4)	0.18	(0.02, 1.64)	0.13
No respiratory viruses	7	(9.0)	1	Ref	-

RSV, respiratory syncytial virus; IQR, interquartile range; RR, risk ratio; MD, mean difference; CI, confidence interval; O2, supplemental oxygen; CPAP, continuous positive airway pressure; WHO, World Health Organization

^a^Risk ratios (or mean differences) estimated from Cox proportional hazards (or linear regression) models adjusted for age, low birth weight, HIV exposure status, severe malnutrition, current breastfeeding, household use of wood as a cooking fuel, WHO disease severity, and oxygen saturation <90% (on room air) at enrollment.

^b^Analysis excludes children with severe malnutrition, defined as weight-for-length <-3 standard deviations on WHO growth curves, mid-upper arm circumference <115mm (for children ≥6 months), or bilateral edema of nutritional origin.

^c^No deaths occurred among the *n* = 18 children with RSV and non-RSV virus coinfection.

### Case-Control Study

We matched a control subject to 133 of 310 (43%) pneumonia cases (Table 1, columns 3–10). Median [IQR] ages of matched case and control subjects were 7.0 [3.0–13.3] and 6.4 [4.0–12.2] months, respectively. Compared with controls, matched cases had a higher rate of HIV infection or exposure (*P* = 0.001), lower maternal education levels (*P* = 0.01), and lived in households that were less likely to have electricity (*P* = 0.01), a municipal or private water source (*P* = 0.01), or a refrigerator (*P* = 0.004), and more likely to use wood as a cooking fuel (*P* = 0.03). Ninety-three of 133 (70%) cases and 45 of 133 (34%) matched controls had one or more respiratory viruses (Table 2, columns 3–10). Among controls, a respiratory virus was detected from 31 of 66 (47%) controls with URI symptoms and 14 of 67 (21%) controls without URI symptoms. Only RSV (*P*<0.0001) and hMPV (*P* = 0.001) were significantly more frequent among cases than controls, although influenza and parainfluenza viruses also tended to be more common among children with pneumonia. Virus-virus coinfections were more frequent among cases than controls (5% vs. 1%, *P* = 0.03), with RSV-rhinovirus accounting for 16 of 25 (64%) coinfections. Only E/R was detected from controls without current URI symptoms.

### Seasonality of Pneumonia Cases

**Fig 1:**
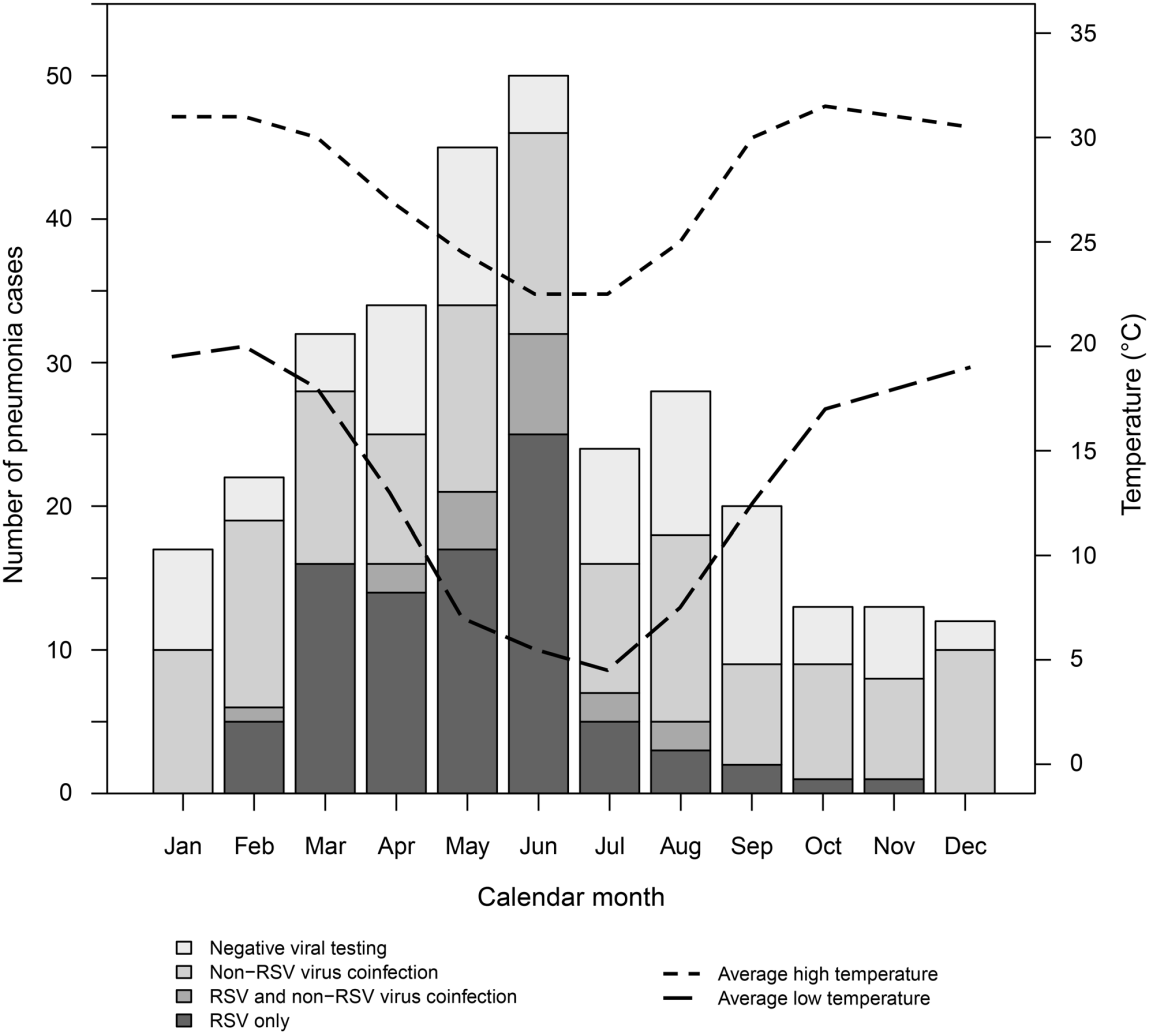
Seasonal distribution of respiratory viruses detected from children 1 to 23 months of age with pneumonia in Botswana, April 2012 to August 2014.

In Fig 1, the detection of respiratory viruses in children with pneumonia is shown by month of enrollment. Detection of RSV varied substantially by season, with the virus being identified from >45% of cases with pneumonia from March to June and <30% of cases for the remainder of the year. The increase in pneumonia enrollments during the early winter months coincided with a peak in RSV activity.

## Discussion

We identified a potential viral etiology in 75% of young children with pneumonia in Botswana. Detection of RSV in pneumonia was associated with worse hospital outcomes but lower in-hospital mortality than detection of non-RSV viruses. RSV and hMPV were the only viruses that were detected more frequently in pneumonia cases than in control children.

RSV was previously reported to be associated with a protracted illness course among children with severe ALRI in developed countries [21,22]. In 136 children hospitalized for ALRI in Sweden, RSV predicted an increased need for supplemental oxygen and a longer length of stay than non-RSV viruses [21]. Similarly, hospitalized children with RSV ALRI in the United States had a more severe course than children with negative viral testing [22]. Few data are available from LMICs, but respiratory virus detection was not independently associated with treatment failure or death in a hospital-based study of Kenyan children [19]. Notably, RSV and non-RSV viruses were not considered separately in multivariable analyses, although treatment failure rates did not significantly differ in bivariable analyses comparing children with RSV, non-RSV viruses, and negative viral testing [19]. Moreover, several studies in Africa previously suggested that the case fatality rate for severe RSV ALRI among HIV-negative children is low [23,24]. However, this study is, to our knowledge, the first detailed examination of associations between detection of specific respiratory viruses and outcomes of severe childhood ALRI in LMICs. We performed analyses adjusted for age, illness severity, and several other potential confounding factors that are prevalent among children in sub-Saharan Africa, including severe malnutrition and HIV infection or exposure [17–19]. Our findings indicate that RSV is an independent risk factor for clinical non-response at 48 hours and is associated with more days of respiratory support and a longer length of stay than non-RSV viruses. However, detection of RSV reduced the risk of in-hospital mortality by 90% compared with non-RSV viruses. In-hospital mortality for RSV ALRI also tended to be lower than for children with negative viral testing, in whom bacterial pneumonia is presumably more likely.

Although these are the first published data on the viral causes of ALRI in Botswana, our findings are similar to those reported in other African countries. Respiratory viruses were detected by PCR in 60% of 805 Kenyan children hospitalized for pneumonia, with RSV and rhinoviruses being the most frequently identified [5]. In rural Mozambique, rhinoviruses, adenovirus, and RSV were detected in 41%, 21%, and 11% of children hospitalized for pneumonia [8]. Rhinoviruses and RSV were also the most frequently identified viruses in studies of pediatric respiratory infections conducted in South Africa [25] and Burkina Faso [4]. Moreover, despite a semi-arid climate, circulation of respiratory viruses was strongly seasonal with the timing of RSV season coinciding with what was recently reported in South Africa [9].

Several prior studies examined associations between detection of specific respiratory viruses by PCR and pediatric ALRI. RSV, influenza A, parainfluenza types 1 and 3, and hMPV were more common in Nepalese children seeking outpatient care for ALRI than in age-matched controls [2]. Similar findings were observed in a study conducted in Alaska that compared children hospitalized for ALRI with asymptomatic controls [26]. However, in two studies conducted in Kenya, only RSV was significantly more frequent in hospitalized children with pneumonia than in outpatient controls [5,6]. A major strength of our study is that controls were matched to cases by enrollment date and primary care clinic. Although this meant that we enrolled a control in fewer than half of cases, this strategy accounted for the seasonality of respiratory virus circulation and ensured that cases and controls were closely matched on location of residence within the Gaborone area. The relatively low rate of detection of influenza and parainfluenza viruses in our cohort limited the comparisons for these viruses, but our results offer further evidence of the potential for both RSV and hMPV to cause severe ALRI. Finally, rhinovirus was frequent among cases, but equally frequent in controls. As rhinovirus can be associated with asymptomatic infection, URI, and ALRI, the case-control methodology we used may be limited for proving ALRI causality. Further community-based, prospective studies are needed to delineate the role of rhinovirus in ALRI.

Although detection of respiratory viruses by PCR may provide valuable prognostic information, there are little data that these assays alter clinical management. Antibiotic use was reduced in some [3,27] but not all pediatric studies [22,28,29], while the effect on length of stay and hospital costs has also been variable [27,28,30]. Additional research on the cost-effectiveness of these assays is needed before use in individual patient management in LMICs can be recommended.

Our study has several limitations. First, it was conducted at a single hospital in Botswana, and the viral etiologies and outcomes of severe ALRI may differ in other settings. Secondly, we did not have a sufficient sample size to assess outcomes for individual viruses other than RSV, choosing instead to analyze non-RSV viruses collectively. Moreover, although outcomes for RSV and non-RSV virus coinfections were broadly similar to those with RSV only, the relatively few viral coinfections in our cohort precluded a formal comparison of outcomes in these groups. Prior studies examining disease severity or outcomes of RSV and non-RSV virus coinfections have yielded conflicting results [31–33]. Finally, testing for bacterial pathogens was not available, and it is possible that the varied outcomes of RSV and non-RSV viruses relate to differential rates of bacterial coinfection. However, data from developed countries indicate that the prevalence of concurrent bacterial infection in infants hospitalized with RSV ALRI is low (<2%) in the absence of respiratory failure [34,35].

In conclusion, respiratory viruses were detected from a substantial proportion of severe ALRI episodes among infants and young children in Botswana. Detection of RSV by PCR may help clinicians in LMICs identify children with severe ALRI who are likely to have a protracted illness course but are at low risk for death. Further research is warranted to determine whether this diagnostic testing might improve clinical management and is cost-effective in settings with limited resources.
